# Maternal depression and malnutrition in children in southwest Uganda: a case control study

**DOI:** 10.1186/s12889-015-2644-y

**Published:** 2015-12-28

**Authors:** Scholastic Ashaba, Godfrey Zari Rukundo, Florence Beinempaka, Moses Ntaro, John C. LeBlanc

**Affiliations:** Department of Psychiatry, Mbarara University of Science and Technology, Mbarara, Uganda; Department of Nursing, Mbarara University of Science and Technology, Mbarara, Uganda; Department of Community Health, Mbarara University of Science and Technology, Mbarara, Uganda; Department of Paediatrics, Psychiatry, Community Health and Epidemiology, Dalhousie University, Halifax, Canada

**Keywords:** Maternal depression, Malnutrition, Children under five years

## Abstract

**Background:**

Malnutrition remains one of the most significant child health problems in developing countries with an estimated 53 % of child deaths per year attributed to being underweight. The 2011 Uganda Demographic and Health Survey (UDHS) showed that 38 % of the children were stunted and 16 % were underweight. While dietary and environmental factors are known major contributors to children's nutritional status, maternal depression may also contribute since it disrupts the mothers’ ability to cope with demands of childcare. This study aimed to determine the association between maternal depression and malnutrition in children aged one to 5 years in southwest Uganda.

**Methods:**

The study was undertaken between October and December 2014 on children aged one to 5 admitted to the Mbarara regional referral hospital. Cases were malnourished children and controls were children with other chronic conditions but normal nutritional status admitted to the same hospital. Children’s ages were recorded, weight and height taken and converted into height for age, weight for height and weight for age and malnutrition was determined based on WHO child growth standards. Mothers of both groups of children were assessed for depression using the depression module of the Mini International Neuropsychiatric Interview (MINI). Participants provided informed consent prior to enrollment. The study was approved by Mbarara University of Science and Technology Research Ethics Committee and funded by MicroResearch.

**Results:**

All 166 mothers who were approached agreed to participate in the study. The prevalence of depression among mothers of malnourished children (86 cases) was 42 % compared to 12 % among mothers of controls (86 controls). The mean age was 25 years (SD 4.43, range 18–40 years). The majority (75 %) were married and most were peasant farmers (62 %). Maternal depression was significantly associated with malnutrition in children with a crude odds ratio of 2.23 (1.08–1.89) and an adjusted odds ratio of 2.4 (1.11–5.18).

**Conclusion:**

Maternal depression impacts negatively on child nutrition and development as shown by a higher prevalence of depression among mothers of malnourished children compared to the control group. Routine screening and treatment for depression should be included in all maternal and child health clinics.

## Background

Malnutrition remains one of the most significant child health problems in developing countries with an estimated 53 % of child deaths per year being attributed to being underweight. Globally, it is estimated that there are nearly 20 million children who are severely and acutely malnourished [1] and most of these children live in south Asia and Sub-Saharan Africa. Child growth studies in 139 countries revealed that 20 % of children below 5 years in low-income and middle-income countries were malnourished [2]. In addition, WHO estimates indicate that approximately 16 % of children from developing countries are severely malnourished basing on a cut off for weight for height, weight for age, and height for age of below −3 standard deviations (−3SD) of the WHO child growth standards [3].

Malnutrition impairs physical growth, increases morbidity and mortality and reduces cognitive development, fertility and physical work capacity [4]. Malnutrition is also an underlying risk factor that increases the morbidity and mortality of many diseases in children and adults [5]. More than 200 million children under the age of 5 years fail to reach their growth and cognitive potential due to lack of adequate nutrition and deficient care [6].

Although dietary and environmental factors play a major role in children's nutritional status [7], maternal mental health has been shown to play a major contributing role [8], possibly by interfering with the mother’s responsibility for child care [9]. Poor maternal mental health, in particular maternal depression has been pointed out as a risk factor for poor growth in young children [10] and infants of mothers with depression have been found to be at a greater risk of growth failure than are infants whose mothers are not depressed [11, 12]. In Uganda, the prevalence of depression in women is estimated to be 27-31 % [13]. A study by Rahman and colleagues [10] estimated that reductions in the prevalence of maternal depression could lead to a reduction in impaired child growth of up to 30 %. Sadly, maternal mental health is still largely neglected as a component in child health programs in developing countries. The much touted integrated management of childhood illness strategy by World Health Organization does not even mention maternal mental health [14].

There are no published studies in Uganda about a possible association between maternal depression and malnutrition despite national surveys indicating that malnutrition in children under five is very rampant especially in southwest Uganda [15]. The main aim of this study was to assess the relationship between maternal depression and malnutrition in children aged one to 5 years in southwest Uganda.

## Methods

We undertook a case–control study between October and December 2014 at the Mbarara Regional Referral Hospital in southwest Uganda.

### Inclusion and exclusion criteria

Children were considered for the study if they were aged one to 5, their mothers were at least 18 years old and their mothers were the primary caregiver of the child during the admission. Cases had malnutrition as defined in the World Health Organization (WHO) child growth standard measurements [16]. The exclusion criterion for cases was a significant comorbid illness identified through investigations done while in hospital. Inclusion criteria for controls were being admitted for a chronic physical illness but having normal nutritional status. Controls were matched to cases according to the child’s age (within 6 months) and gender.

### Measurements

#### Children anthropometry

Children’s weight, height and mid upper arm circumference (MUAC) were taken following standard anthropometric procedures [17] and their ages were determined by maternal report. Anthropometric measures were converted into height for age, weight for height and weight for age and children were considered to be malnourished if their Z scores were below minus three standard deviations (−3SD) and had a MUAC of less than 115 mm according to WHO growth standard measurements [16].

### Depression among mothers

Following consent, the mothers of both cases and controls were assessed for current depression using the depression module of the Mini International Neuropsychiatric interview (M.I.N.I.) [18]. The MINI is a brief structured interview for major axis I Psychiatric disorders in DSM-IV and ICD-10 [18] that can be administered in about 15 minutes by a clinician after a brief training. The depression module of the Mini International neuropsychiatric interview [18] was selected because it is superior to screening tools that have been used in other similar studies [19–21]. Moreover, the depression module of the MINI has been translated into local languages and used in different parts of Uganda in different contexts [22–25]. We asked mothers two screening questions: 1) feeling sad most of the day almost every day for two weeks, and 2) loss of interest in activities previously enjoyed. If a participant answered yes to one of these, more questions about depression symptoms were asked including change in appetite, trouble sleeping, feeling tired most of the time, feeling worthless, difficulty with concentration and having suicidal feelings. If a participant answered yes to five or more of the above questions they were considered depressed. Two trained psychiatric nursing officers did data collection. Participants who screened positive for depression were referred for treatment in the hospital’s mental health unit.

### Sample size determination

Sample size was determined using OpenEpi software version 3.03.17 assuming a two-sided significance level (alpha or α) of 0.05 and a power of 80 %, and assuming that 20 % of controls had depression compared to 40 % of cases. This gave us a total sample of 83 cases and 83 controls.

### Ethical approval and funding

The study was approved by the Research and Ethics Committee (REC) of Mbarara University of Science and Technology and funded by a MicroResearch grant (www.microresearch.ca). We sought informed consent from the participating mothers.

### Data analysis

Data were inspected and data entry errors were removed. Data were analyzed using SPSS version 18. Binary variables were summarized as percentages of cases and controls with the attribute (e.g., depressed yes/no). We analyzed group differences on variables using a chi square test. Odds ratios (OR) and 95 % confidence intervals (CI) were determined using logistic regression analysis with the dependent variable being case/control status and the independent variables being mother depressed at the time of admission, age, marital status, education attained, occupation, source of income and number of children. Results were considered significant if the p-value was ≤ 0.05. First, we used bivariate logistic regression analysis to estimate OR for each variable. Those that demonstrated an association with the outcome were inserted into a multiple logistic regression model to estimate the adjusted OR of association between maternal depression and malnutrition in children.

## Results

Eighty-three cases and 83 controls were recruited between October and December 2014. The prevalence of depression was 42 % among mothers of malnourished children (cases) compared to 12 % among mothers of controls. The average age of the participants was 25 years (SD 4.4, range 18–40 years). The majority of the mothers (75 %) were married and 63 % were peasant farmers. There were no statistically significant differences between controls and cases (Table 1). Of the malnourished children 75 % were stunted (low height for age), 84 % were wasted (low weight for height) while 87 % were underweight (low weight for age) (Table 2). Bivariate logistic regression analysis showed a strong association between malnutrition in children and maternal depression (Table 3). This remained significant in a multivariate logistic regression OR 2.4 (1.11–5.18) (Table 4) that also included all other covariates. This study also found an association between low maternal education and malnutrition in children one to five years of age during bivariate analysis but this association was explained away by other factors in the multivariate model.

**Table 1:** Socio-demographic characters of the mothers (cases and controls)

Variables	Malnutrition in children		Total (%)	
	Cases (*n* = 83)	Controls (*n* = 83)		X^2^ (*p*-value)*
	Proportion (%)	Proportion (%)		
Mother’s age				
18–24	41 (25 %)	36 (22 %)	77 (47 %)	1.29 (0.53)
25–30	26 (16 %)	33 (20 %)	59 (36 %)	
> 30	16 (10 %)	14 (8 %)	30 (18 %)	
Mother’s marital status				
Married	60 (36 %)	65 (39 %)	125 (75 %)	0.81 (0.37)
Single/Separated/widowed	23 (14 %)	18 (11 %)	41 (25 %)	
Mother’s level of education				
Lower primary (p1-p4)	33 (20 %)	19 (11 %)	52 (31 %)	5.53 (0.06)
Upper primary (p5-p7)	35 (21 %)	46 (28 %)	81 (49 %)	
High school or more	15 (9 %)	18 (11 %)	33 (20 %)	
Mother’s occupation				
Peasant farmer	57 (34 %)	46 (28)	104 (63 %)	4.27 (0.12)
Retail business	13 (8 %)	24 (15 %)	37 (22 %)	
Casual labor/no occupation	13 (8 %)	12 (7 %)	25 (15 %)	
Mother’s source of income				
Farming	48 (29 %)	37 (22 %)	85 (57 %)	3.70 (0.16)
Retail business	21 (13 %)	32 (19)	53 (32 %)	
Casual laborer/no source of income	14 (5 %)	14 (5 %)	28 (10 %)	
Number of children				
1–2	44 (27 %)	43 (26 %)	87 (53 %)	0.40 (0.82)
3–4	26 (16 %)	24 (14 %)	50 (30 %)	
>4 children	13 (9 %)	16 (10 %)	29 (18 %)	

* = *p* ≤ 0.05

**Table 2:** Anthropometric measurements of children (cases)

Variables	(*N* = 83) proportion	Percentage (%)
Stunted (below −3SD height for age)	62	75 %
Underweight (below −3SD weight for age)	72	87 %
Wasted (below −3SD weight for height)	69	83 %

**Table 3:** Maternal depression and other socio-demographic factors associated with malnutrition in children

Variables	Unadjusted OR (95 % CI)	*P*-value*
Depression in the mother		
Depression	2.4 (1.18–4.79)	0.015^*^
Age of the mother (years)		
18–24^a^		
25–30	1.2 (0.61–2.52)	0.558
> 30	1.2 (0.50–2.91)	0.680
Level of education of the mother		
Lower Primary (p1-p4)^a^		
Upper Primary (p5-p7)	2.4 (1.48–7.61)	0.004
Secondary school (s1-s6)	2.4 (0.89–6.45)	0.084
Marital status		
Married^a^		
Single/separated/widowed	0.9 (0.43–1.89)	0.799
Occupation of the mother		
Peasant farmer^a^		
Retail business	1.6 (0.76–3.54)	0.209
Casual laborer/no occupation	1.2 (0.49–3.02)	0.683
Mother’s Source of income		
Farming^a^		
Retail business	2.0 (0.73–5.47)	0.177
Casual labor/no source of income	2.6 (0.90–7.48)	0.076
Number of children		
1–2^a^		
3–4	0.9 (0.40–1.81)	0.688
> 4	1.9 (0.79–4.38)	0.152

*p ≤ 0.05

^a^Reference categories

**Table 4:** Adjusted factors associated with malnutrition in children

Variable	Adjusted OR (95 % CI)	*P*-value*
Maternal depression	2.4 (1.11–5.18)	0.03^*^
Age of the mother (years)		
18–24^a^		
25–30	0.9 (0.36–2.44)	0.88
> 30	0.6 (0.15–2.54)	0.50
Mothers’ level of education		
Lower primary (p1-p4)^a^		
Upper primary (p5-p6)	0.5 (0.15–1.34)	0.15
Secondary education (s1-s6)	1.3 (0.50–3.30)	0.59
Marital status		
Married^a^		
Single/separated/widowed	1.4 (0.60–3.20)	0.44
Occupation of the mother		
Peasant farmer^a^		
Retail business	1.1 (0.18–6.54)	0.93
Casual laborer/no occupation	1.3 (0.29–6.08)	0.72
Source of income		
Farming^a^		
Retail business	2.3 (0.64–8.30)	0.20
Casual labor/no source of income	2.2 (0.46–10.2)	0.33
Number of children		
1–2^a^		
3–4	1.2 (0.42–3.25)	0.77
> 4	2.7 (0.72–10.4)	0.41

*p ≤ 0.05

^a^Reference categories

^*^significant values

## Discussion

For the first time in an African setting, we have demonstrated an association between maternal depression and malnutrition in children aged one to 5. This confirms findings in other low and high-income countries including studies in Jamaica, Pakistan, India and the United Kingdom [11, 19–21, 26, 27]. Infants of depressed mothers are likely to be underweight and stunted compared to children of mothers without depression [20, 21, 28]. The association between maternal depression and malnutrition persisted after controlling for education, family size, and source of income.

Maternal depression may contribute to under nutrition in children by having a negative impact on interpersonal behaviour and parenting as well as impairment in social functioning associated with most psychiatric disorders [29]. Maternal depression not only reduces maternal interest in a child [27] but also impairs a woman’s ability to cope with the responsibilities of being a mother. This leads to an inability to provide a healthful diet for the child [30]. Maternal depression reduces a mother’s involvement in maternal care, which impairs physical growth of the child [20, 31]. Mothers with depression tend to be less responsive towards their children [29] and are less likely to form a secure attachment with their children [32] which may also contribute to neglect of maternal nurturing roles.

This study found no association between maternal level of education and malnutrition in children one to five years. This is inconsistent with other studies [28, 33, 34]. Gwatkin and colleagues found high levels of malnutrition among children of less educated women in developing countries [33] while Reed and colleagues found higher level of education to be associated with improved child weight for age in Benin, West Africa [34]. The studies that have found an association between maternal low level of education and malnutrition in children indicate that mothers with low education tend to live in rural areas with more limited nutritional knowledge and health access [35]. It has been argued that education empowers women, influencing their capacity to make decisions [28] concerning children’s nutrition and access to health services [36]. Educated women are able to make better health choices for their children as well as having more knowledge about good child nutrition and feeding practices [37].

Our study has limitations. It was a hospital-based study making it difficult to generalize the results to the general population. The study was cross sectional and hence it is difficult to tease out the cause –effect relationship between maternal depression and malnutrition in children.

However, given that, the control children had chronic illness but not malnutrition helps mitigate that bias. That 12 % percent of these control mothers had depression is not surprising but this was much less than the 42 % amongst mothers of hospitalized malnourished children. The fact that the study was carried out in the hospital setting could have contributed to emotional distress associated with the children’s sickness in both groups. A survey in the community in southwestern Uganda would be very important to see if the same association exists.

## Conclusion

This study shows that maternal depression in mothers of young children is very common and is linked to malnutrition. All maternal newborn and childcare comprehensive programs aimed at improving health outcomes need to address this important mental illness for its own sake as well as the likely outcome that depressed mothers will provide inadequate care and diets for their children.
